# Novel *RAI1*:c.2736delC Variant in Smith–Magenis Syndrome: Identification by Whole Genome Sequencing and Joint Analysis

**DOI:** 10.3390/jpm14090901

**Published:** 2024-08-25

**Authors:** Mario Cuk, Busra Unal, Nives Jandric, Connor P. Hayes, McKenzie Walker, Feruza Abraamyan, Kristina Crkvenac Gornik, Arezou A. Ghazani

**Affiliations:** 1 Department of Pediatrics, School of Medicine, University Hospital Centre Zagreb, University of Zagreb, 10000 Zagreb, Croatia; 2 Division of Genetics, Brigham and Women’s Hospital, Boston, MA 02115, USA; 3 Department of Laboratory Diagnostics, Division of Cytogenetics, University Hospital Centre Zagreb, 10000 Zagreb, Croatia; 4 Department of Medicine, Brigham and Women’s Hospital, Boston, MA 02115, USA; 5 Harvard Medical School, Boston, MA 02115, USA

**Keywords:** Smith–Magenis syndrome, retinoic acid-induced 1 protein, neurodevelopmental disorders, joint whole-genome sequencing analysis

## Abstract

Smith–Magenis syndrome is a complex neurobehavioral genetic disorder with a broad phenotypic spectrum. While the etiology of SMS is commonly attributed to one-copy interstitial deletion in the 17p11.2 region (90–95% of cases), variants identified by sequence analysis in *RAI1* have also been reported in 5–10% of cases. In this study, we report a 9-year-old male with global cognitive and psychomotor developmental delay, musculoskeletal and cardiovascular abnormalities, and dysmorphic craniofacial features. Joint analysis was performed on the whole-genome sequencing data obtained from the proband, unaffected parents, and unaffected brother. This quad analysis identified the novel de novo *RAI1*:c.2736delC variant. This is the first report of this variant in the literature. This report highlights the details of genome analysis and the patient’s phenotypic spectrum.

## 1. Introduction

Smith–Magenis syndrome (SMS; OMIM #182290) is a complex neurobehavioral disorder with an estimated prevalence of 1:15,000. This rare syndrome is characterized by neurodevelopmental delay, coarse facial features evolving with age, and maladaptive behaviors that include prolonged temper tantrums and self-injurious behaviors, including hand-biting, trichotillomania, and polyembolokoilamania [1]. During infancy, hypotonia, hyporeflexia, and feeding difficulties are commonly seen as neurological findings [2,3,4,5]. The maladaptive behavior phenotype is typically recognized after 18 months of age. The classic neurological features include intellectual disability, delayed speech, sensory integration problems, decreased pain sensitivity, and abnormalities in sleep patterns [1]. Additional system involvements, such as musculoskeletal and cardiovascular, are also reported as part of SMS [2,3,4,5,6].

The underlying genomic etiology of SMS is interstitial deletion at 17p11.2 in 90–95% of patients. This genomic location encompasses the retinoic acid-induced 1 (*RAI1*) gene. *RAI1* is the key gene in SMS and plays a regulatory role in healthy skeletal and nervous system development, behavioral maturation, and circadian rhythm. Pathogenic de novo single nucleotide variants (SNVs) in *RAI1* are reported in 5–10% of patients with SMS [2,5,6,7]. Most reported *RAI1* variants are missense, nonsense, and splice site variants [4]. The diagnosis of the SMS syndrome is based on clinical molecular diagnostics tests. The first-tier molecular test is chromosomal microarray analysis (CMA), and if negative, a sequencing-based test is typically performed. Genome studies have reported SMS-like features, including ID, sleep disturbances, and self-injuries behaviors in patients without an SMS molecular diagnosis who harbored deleterious variants in *KMTD2*, *MECP2*, *KDM5C, IQSEC2*, and *DEAF1* genes [8,9].

Here, we report a 9-year-old male with global cognitive and psychomotor developmental delay and facial, musculoskeletal, and cardiovascular abnormalities. The patient’s chromosomal microarray and karyotype tests were normal. The joint analysis of whole-genome sequencing (WGS) data detected a pathogenic novel de novo variant. This report highlights the importance of sequencing analysis in patients clinically suspected of SMS, with negative findings on conventional diagnostic tests. Sharing the genotype and detailed phenotype of these relatively rare SMS cases helps understand the genetic and phenotypic spectrum of the disease.

## 2. Material and Methods

### 2.1. Participants

The index patient, a 9-year-old male, his parents, and his younger brother were enrolled in the CROseq Genome Program. The CROseq Genome Program is a collaborative research program between Brigham and Women’s Hospital (BWH) (Boston, MA, USA) and the Department of Pediatrics, University Hospital Center Zagreb (Zagreb, Croatia), supported by the Mila Za Sve Foundation (Rijeka, Croatia). This program aims to investigate the genomic etiology of disease in patients with complex phenotypes and negative conventional genetic tests. Patient consent, enrollment, and clinical assessment were performed at the University Hospital Center Zagreb.

### 2.2. Clinical Assessment of Brain Structure and Activity

The brain abnormalities were assessed by MRI scanning (MAGNETOM Trio, A Tim System 3T eco, Zagreb, Croatia). The patient was under general anesthesia during the scan. Age-adjusted repetition time and time-to-echo were applied to create multiplanar T1- and T2-weighted images. The electroencephalogram (Nihon Kohden, Neurofax EEG-1200K; Zagreb, Croatia) examination was performed to evaluate the brain activity. During the record, the sampling rate was 200–10,000 Hz. The patient’s forehead was cleaned with alcohol to lower the impedance less than 5 Ω for each electrode. The electrodes were set up in FPz-F9 and FPz-AF7 of the international 10–20 system, which focused on the brain in the same hemisphere.

### 2.3. Traditional Genetic Testing

Karyotyping was performed at University Hospital Center Zagreb according to standard procedures. Briefly, the lymphocytes were purified from the peripheral blood sample. Cells were arrested at metaphase and fixed before being harvested for slide preparation. The number and morphology of chromosomes were assessed in at least 20 metaphases. The karyotype result was reported per the International System for Human Cytogenomic Nomenclature (ISCN) 2020.

Chromosome microarray (CMA) was performed at University Hospital Center Zagreb according to standard procedures. Briefly, genomic DNA was isolated with the FlexiGene DNA Kit (Qiagen), and CMA was performed using Agilent 4 × 180k aCGH+SNP array (Agilent Technologies, Santa Clara, CA, USA). The genomic DNA of the test and control samples were labeled with fluorescent dyes and hybridized to the array. The array was scanned with a DNA Microarray Scanner (Agilent Technologies, Santa Clara, CA, USA). Data were obtained using the “CytoGenomics 5.1.1.15” software (Agilent Technologies, Santa Clara, CA, USA) to generate the final plots.

### 2.4. Sample Preparation and Whole-Genome Sequencing (WGS)

DNA extraction and WGS were performed at the Medical College of Wisconsin (Milwaukee, USA). Genomic DNA was isolated from peripheral blood samples (2 mL) with a purity ratio of 1.75–2.0. After robotic DNA library construction, sequencing was conducted on the NovaSeq 6000 platform, with an average depth of 40×. 

### 2.5. Quad Joint Whole-Genome Analysis

Genome analyses were performed at the BWH (Boston, MA, USA). The quad analysis was performed on the WGS data obtained from all available family members. This included the affected proband, the unaffected parents, and the unaffected proband’s brother. Following the quad analysis, a phenotype-based analysis was performed using human phenotype ontology (HPO) terms of the patient’s indications. Variants were prioritized by the gene’s association with the phenotype and variant type. The HPO terms were: Neurodevelopmental delay (HP:0012758), moderate intellectual disability (HP:0002342), delayed speech and language development (HP:0000750), attention deficit hyperactivity disorder (HP:0007018), bicuspid aortic valve (HP:0001647) and stenosis (HP:0001650), failure to thrive (HP:0001508), abnormal facial shape (HP:0001999), unilateral ptosis (HP:0007687), frontal bossing (HP:0002007), relative macrocephaly (HP:0004482), abnormality of mitochondrial metabolism (HP:0003287).

All variants across the genome were included in the investigation. Following the technical assessment, medium, high, and very high-quality variants were further evaluated, and low-quality variants were excluded. The variant frequencies were annotated by gnomAD exome and genome population allele frequencies. The in silico prediction algorithms for missense variants included CADD, REVEL, Polyphen, SIFT, MutationTaster, Mutation Assessor, FATHMM, FITCONS, GENOCANYON, dbscSNV ADA, and dbscSNV RF. The SpliceAI prediction score was used for the evaluation of splice variants.

Zygosity analysis was performed to identify and evaluate compound heterozygous and homozygous variants in the proband. This included variants in the proband that resided in genes with autosomal recessive inheritance (related to the patient’s HPOs) and were inherited from each unaffected parent. Separately, de novo analysis was performed to assess related variants present in the proband but absent in the unaffected parents and the brother.

The variant classification was performed according to ACMG-AMP guidelines (25741868). For the PM2 and BS1 criteria, the gene-specific threshold was applied based on gnomAD (v2.1.1) aggregated allele frequency. The threshold for aggregated in silico prediction score was set at >0.7 for a deleterious effect, and <0.15 for a benign effect. The gene metrics of pLI = 1 and/or o/e < 0.35 were used (where pLI denotes the probability of loss-of-function intolerance).

## 3. Results

### 3.1. Clinical Description

The proband is a 9-year-old male presented with global cognitive and psychomotor developmental delay, musculoskeletal and cardiovascular abnormalities, and dysmorphic craniofacial features. During his infancy, the patient had generalized hypotonia and feeding difficulties. His brain ultrasound at four months of age was normal. Following intensive physical therapy, he started walking at two years old. The psychomotor assessment revealed that the patient had severe psychomotor retardation. The brain MRI performed at 3.5 years of age showed perinatal hypoxic-ischemic lesions. The electroencephalogram was unremarkable.

**Figure 1:**
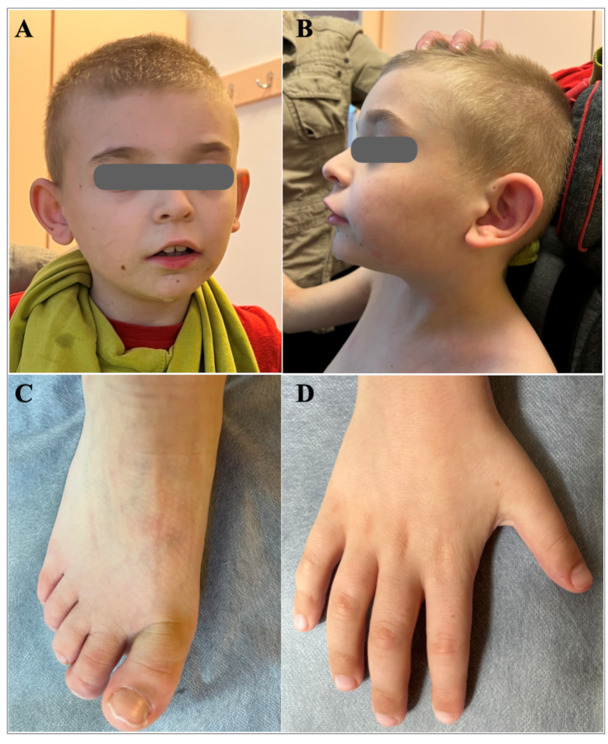
Dysmorphic features of the proband at the age of 9 years. (**A**) Brachycephaly, broad face, hypertelorism, wide nasal bridge, midface retrusion, deeply set ears, tented and down-turned upper lip, everted upper lip vermilion, light-colored hair; (**B**) Brachycephaly, prominent forehead, short and full-tipped nose, deeply set ears, midface retrusion, micrognathia, tented and down-turned upper lip, everted upper lip vermilion, light-colored hair; (**C**) Short foot, brachydactyly; (**D**) Short palm, brachydactyly.

The detailed clinical evaluation at 9 years of age indicated brachycephaly, broad face, hypertelorism, wide nasal bridge, short and full-tipped nose, midface retrusion, deeply set ears, tented and down-turned upper lip, everted upper lip vermilion, micrognathia, and low hairline (Figure 1). He exhibited global cognitive and psychomotor developmental delay, severe ID, and developmental language disorder. The pervasive developmental disorder was evident with the lack of speech development and poor contact. Behavioral phenotypes included aggression toward others, self-injurious episodes, and polyembolokoilamania. He did not acquire sphincter control and exhibited incontinence. He had sleep disturbances, including night awakening, with difficulty falling back asleep. There were skeletal abnormalities, including mild scoliosis, thoracic kyphosis, short palm and foot, brachydactyly, and partial syndactyly of both hands’ second and third fingers (Figure 1). He reportedly had a bicuspid aortic valve with mild stenosis and congenital left eyelid ptosis.

### 3.2. Genomic Findings

#### 3.2.1. CMA and Karyotype Analysis

Karyotype analysis revealed a normal 46, XY result. CMA analysis of the proband detected a maternally inherited 2.1 Mb duplication in 22q11.21 (Supplementary Figures S1 and S2). This region contains OMIM genes *SLC25A1*, *CDC45*, *GP1BB*, *TBX1*, *TXNRD2*, *COMT*, *TANGO2*, *RTN4R*, *SCARF2*, *PI4KA*, *SERPIND1*, *SNAP29,* and *LZTR1*. The mother is unaffected. Therefore, this 22q11.21 gain was considered unrelated to the patient’s clinical findings of SMS. The patient was enrolled in the CROseq Genome Program, and a quad WGS joint analysis was performed.

#### 3.2.2. Quad WGS Analysis

After WGS, joint analysis was performed on sequencing data from the proband, unaffected brother, and parents. HPO-based analysis revealed 10,290 variants in 2567 genes. Compound heterozygous analysis interrogated possible causative variants inherited from the unaffected parents. Quad analysis did not identify any deleterious homozygous or compound heterozygous candidate variants in the proband.

**Figure 2:**
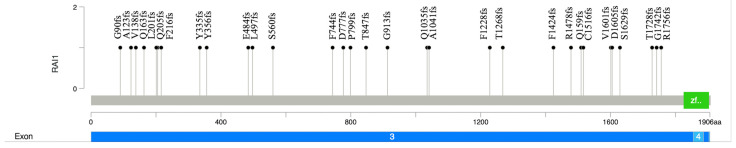
Lollipop plot of the pathogenic and likely pathogenic *RAI1* frameshift variants reported in the literature. The plot shows the p.G913fs variant identified in the proband in this study and the distribution of frameshift *RAI1* variants reported in the ClinVar database to date (n = 30). The Y axis shows the variant number, and the X axis illustrates the position of amino acid residues along the RAI1 protein. It includes exon 3 and exon 4. The green box denotes the PHD-like zinc-binding domain.

De novo analysis interrogated variants related to the HPO that were present in the proband but absent in the parents and the unaffected sibling. This analysis identified the variant NM_030665.4 (*RAI1*):c.2736delC (p.Gly913Alafs*37), located in exon 3 of *RAI1* (Figure 2). This variant is a 1 bp deletion that results in a frameshift and a premature stop codon. Due to its location, this variant likely results in a premature translational stop signal and nonsense-mediated mRNA decay. The gene constraint metrics for *RAI1* are pLI = 1, o/e = 0.04. The variant has not been reported in the gnomAD, ClinVar, or any published database or literature. The phasing analysis by quad WGS confirmed that the parents and younger brother did not harbor this variant. No other deleterious variants in any genes related to the patient’s HPO list were identified.

## 4. Discussion

Smith–Magenis Syndrome, also known as 17p11.2 microdeletion syndrome, was first discovered in 1986 [10]. Since then, the genomic and clinical spectrum of more than 500 SMS patients has been reported [11]. Most cases of SMS are caused by microdeletions in 17p11.2, while 5–10% of cases are due to sequence variants in *RAI1* [2]. When the phenotypic findings suggest SMS, the first molecular testing approach is generally CMA, followed by a single gene or multipanel sequence analysis that includes *RAI1.*

The *RAI1* gene has a regulatory role in embryogenesis through the transcriptional regulation of many genes [12,13,14]. These include the skeletal development genes *PSTPIP2* and *ANGH* [15]; lipid metabolism genes *LIPE*, *HMGCS1*, and *INSIG1* [15]; neurological development genes *ZIC1*, *PSEN2*, *RXRB*, *CLN8*, *SMA4*, *NF1*, and *KMT2A*; behavioral function gene *SCN12A* 19236431 [15]; circadian activity genes *NR1D2*, *PER2*, *PER3*, *CRY1*, and *ARNTL* 22578325; cellular growth and cell cycle regulation genes *SPTBN1*, *POLDIP3*, *PPP1R14D*, *GLI3*, *KMT2A*, and *ADD3* [15]; and insulin regulation genes *INSIG1*, *PIK3R1*, *ZNF236*, and *LIPE* [15]. As a result, patients with SMS commonly have moderate to severe neurodevelopmental and behavioral abnormalities, metabolic problems, sleep problems, and skeletal abnormalities [3].

Here, we report a patient with a spectrum of SMS who harbored the pathogenic and novel NM_030665.4 (RAI1):c.2736delC variant. The variant was de novo and absent in unaffected family members. This variant is in exon 3 (of 6 exons). Most reported pathogenic *RAI1* variants are de novo and are in exon 3 [4], which encodes nearly 98% of the protein. There are reports of mutational hot spots in this exon [16]. The *RAI1*-encoded nuclear protein has a zinc finger homology structural domain, which is indicative of the transcription regulatory activity of *RAI1* [17]. The truncating variants lead to aberrant cytoplasmic subcellular localization and, therefore, the inability of transcription activation [18]. To date, there are 78 frameshift, 29 nonsense, and 4 missense variants reported as pathogenic or likely pathogenic in ClinVar. Most of these variants, including the *RAI1*:c.2736delC variant in our case, are in exon 3 (Figure 2).

The broad phenotypical variation of SMS stems from the genetic pathophysiology of the genomic region. Patients with the 17p11.2 microdeletion are more likely to have severe cognitive impairment, hearing loss, cardiac abnormalities, and hypotonia. These features are associated with the deletion of genes residing within the region [19]. Patients with normal 17p copy number who carry a deleterious sequence variant in *RAI1* reportedly exhibit polyembolokoilamania (inserting foreign objects into orifices), skin picking, and self-hugging behavior [19]. Consistent with these reports, our patient exhibited these behaviors. Our patient also exhibited severe intellectual disability and cardiac abnormalities, as well as hypotonia during infancy. The joint analysis did not reveal any additional alterations in the genome consistent with these phenotypes.

In conclusion, we report a detailed clinical description of a patient with SMS and the novel pathogenic *RAI1*:c.2736delC sequence variant. Reporting clinical findings of SMS and pathogenic *RAI1* variants helps expand the understanding of the genetic pathophysiology of this complex syndrome.

## Data Availability

Data for this manuscript are subject to BWH institutional and GDPR privacy policies and restricted from inclusion in repositories. They may be available upon request.

## Supplementary Materials

The following supporting information can be downloaded at: https://www.mdpi.com/article/10.3390/jpm14090901/s1, Figure S1. Chromosomal microarray of the proband. Figure S2. Chromosomal microarray of the mother.

## References

[1] Elsea S.H., Girirajan S. (2008). Smith-Magenis syndrome. Eur. J. Hum. Genet..

[2] Gropman A.L., Duncan W.C., Smith A.C. (2006). Neurologic and developmental features of the Smith-Magenis syndrome (del 17p11.2). Pediatr. Neurol..

[3] Adam M.P., Feldman J., Mirzaa G.M., Pagon R.A., Wallace S.E., Bean L.J.H., Gripp K.W., Amemiya A. (1993). GeneReviews. https://www.ncbi.nlm.nih.gov/books/NBK1310/.

[4] Rinaldi B., Villa R., Sironi A., Garavelli L., Finelli P., Bedeschi M.F. (2022). Smith-Magenis Syndrome-Clinical Review, Biological Background and Related Disorders. Genes.

[5] Yeetong P., Vilboux T., Ciccone C., Boulier K., Schnur R.E., Gahl W.A., Huizing M., Laje G., Smith A.C. (2016). Delayed diagnosis in a house of correction: Smith-Magenis syndrome due to a de novo nonsense RAI1 variant. Am. J. Med. Genet. A.

[6] Carmona-Mora P., Encina C.A., Canales C.P., Cao L., Molina J., Kairath P., Young J.I., Walz K. (2010). Functional and cellular characterization of human Retinoic Acid Induced 1 (RAI1) mutations associated with Smith-Magenis Syndrome. BMC Mol. Biol..

[7] Slager R.E., Newton T.L., Vlangos C.N., Finucane B., Elsea S.H. (2003). Mutations in RAI1 associated with Smith-Magenis syndrome. Nat. Genet..

[8] Loviglio M.N., Beck C.R., White J.J., Leleu M., Harel T., Guex N., Niknejad A., Bi W., Chen E.S., Crespo I. (2016). Identification of a RAI1-associated disease network through integration of exome sequencing, transcriptomics, and 3D genomics. Genome Med..

[9] Berger S.I., Ciccone C., Simon K.L., Malicdan M.C., Vilboux T., Billington C., Fischer R., Introne W.J., Gropman A., Blancato J.K. (2017). Exome analysis of Smith-Magenis-like syndrome cohort identifies de novo likely pathogenic variants. Hum. Genet..

[10] Smith A.C., McGavran L., Robinson J., Waldstein G., Macfarlane J., Zonona J., Reiss J., Lahr M., Allen L., Magenis E. (1986). Interstitial deletion of (17)(p11.2p11.2) in nine patients. Am. J. Med. Genet..

[11] Wolters P.L., Gropman A.L., Martin S.C., Smith M.R., Hildenbrand H.L., Brewer C.C., Smith A.C. (2009). Neurodevelopment of children under 3 years of age with Smith-Magenis syndrome. Pediatr. Neurol..

[12] Tahir R., Kennedy A., Elsea S.H., Dickinson A.J. (2014). Retinoic acid induced-1 (Rai1) regulates craniofacial and brain development in Xenopus. Mech. Dev..

[13] Bi W., Yan J., Shi X., Yuva-Paylor L.A., Antalffy B.A., Goldman A., Yoo J.W., Noebels J.L., Armstrong D.L., Paylor R. (2007). Rai1 deficiency in mice causes learning impairment and motor dysfunction, whereas Rai1 heterozygous mice display minimal behavioral phenotypes. Hum. Mol. Genet..

[14] Falco M., Amabile S., Acquaviva F. (2017). gene mutations: Mechanisms of Smith-Magenis syndrome. Appl. Clin. Genet..

[15] Girirajan S., Truong H.T., Blanchard C.L., Elsea S.H. (2009). A functional network module for Smith-Magenis syndrome. Clin. Genet..

[16] Dubourg C., Bonnet-Brilhault F., Toutain A., Mignot C., Jacquette A., Dieux A., Gérard M., Beaumont-Epinette M.P., Julia S., Isidor B. (2014). Identification of Nine New RAI1-Truncating Mutations in Smith-Magenis Syndrome Patients without 17p11.2 Deletions. Mol. Syndromol..

[17] Bi W., Saifi G.M., Shaw C.J., Walz K., Fonseca P., Wilson M., Potocki L., Lupski J.R. (2004). Mutations of RAI1, a PHD-containing protein, in nondeletion patients with Smith-Magenis syndrome. Hum. Genet..

[18] Carmona-Mora P., Canales C.P., Cao L., Perez I.C., Srivastava A.K., Young J.I., Walz K. (2012). RAI1 transcription factor activity is impaired in mutants associated with Smith-Magenis Syndrome. PLoS ONE.

[19] Girirajan S., Vlangos C.N., Szomju B.B., Edelman E., Trevors C.D., Dupuis L., Nezarati M., Bunyan D.J., Elsea S.H. (2006). Genotype-phenotype correlation in Smith-Magenis syndrome: Evidence that multiple genes in 17p11.2 contribute to the clinical spectrum. Genet. Med..

