# Supplementary material for: Novel RAI1:c.2736delC Variant in Smith–Magenis Syndrome: Identification by Whole Genome Sequencing and Joint Analysis
Source: J Pers Med. 2024 Aug 25;14(9):901. doi: 10.3390/jpm14090901 (PMC11432845; doi:10.3390/jpm14090901)
Supplement: Supplementary file 1 [file jpm-14-00901-s001.zip › jpm-3146112-supplementary.pdf]

Figure-Proband 1P CMA.

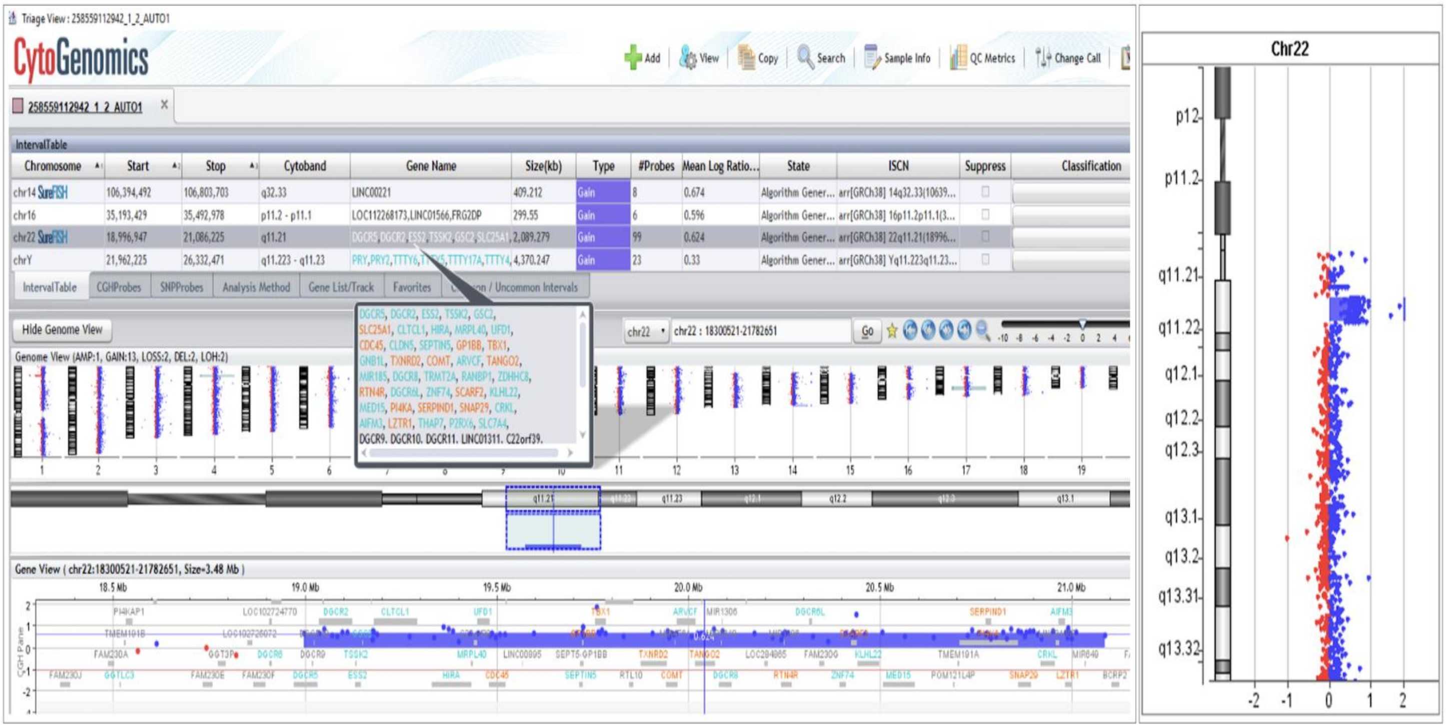

Supplementary Figure S1. Chromosomal microarray of the proband. Interstitial duplication of the long arm of chromosome 22 in the region 22q11.21 (18996947_21086225), 2.1 Mb in size (arr[GRCh38] 22q11.21(18996947-21086225)x3 mat).

Figure-Mother 2M CMA.

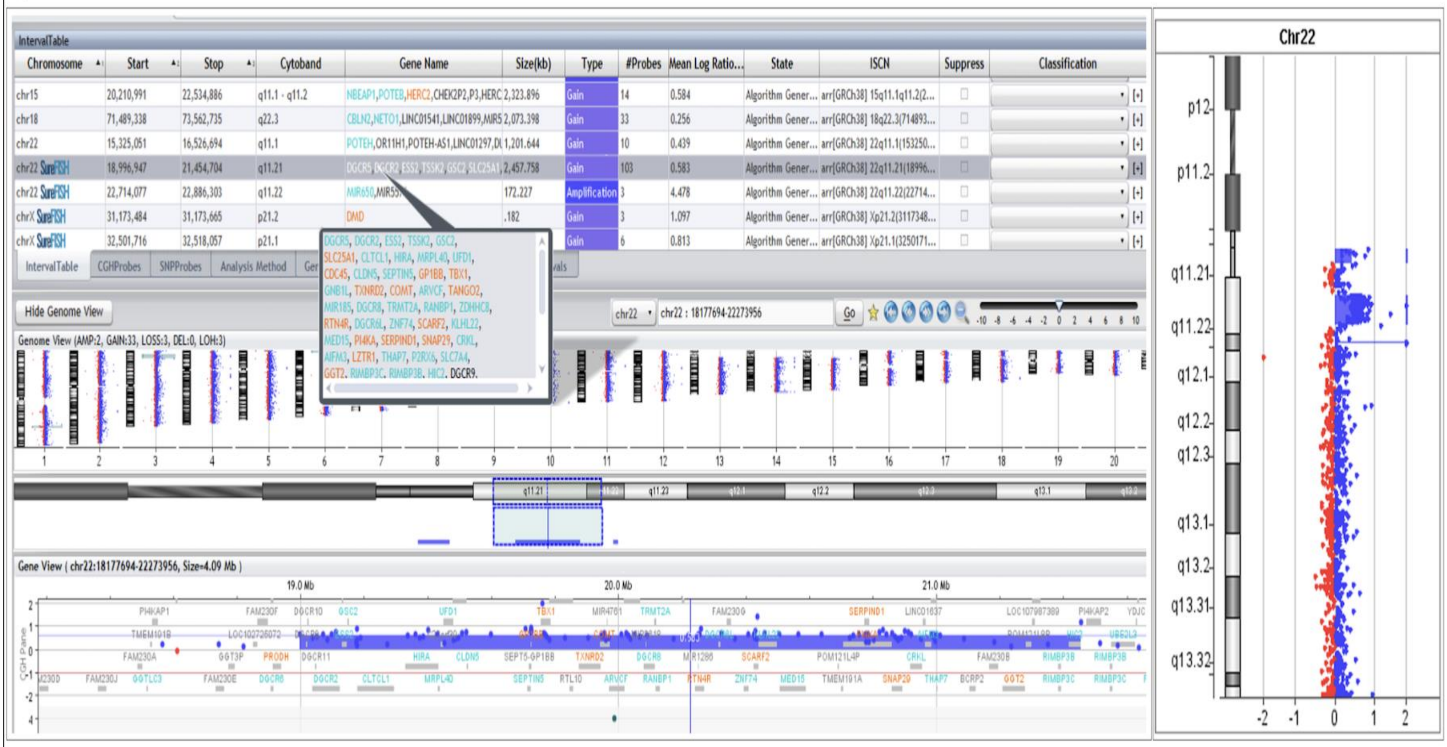

Supplementary Figure S2. Chromosomal microarray of the mother. Interstitial duplication of the long arm of chromosome 22 in the q11.21 region (18996947-21454704), 2.46 Mb in size (arr[GRCh38]22q11.21 (18996947_21454704)x3.
